# Adaptation of in vitro methodologies to estimate the intestinal digestion of lipids in ruminants

**DOI:** 10.1093/tas/txab135

**Published:** 2021-08-17

**Authors:** James R Vinyard, Efstathios Sarmikasoglou, Sarah L Bennett, Jose A Arce-Cordero, Glen Aines, Kari Estes, Antonio P Faciola

**Affiliations:** 1 Department of Animal Sciences, University of Florida, Gainesville, FL, USA; 2 Balchem Corporation, New Hampton, NY, USA

**Keywords:** bile, lipase, pancreatin, rumen protection, three-step procedure

## Abstract

The objective of this study was to adapt existing in vitro methodologies to determine the extent of intestinal digestion of corn oil (CO), canola oil (CA), and beef tallow (BT) via manipulation of incubation length and concentrations of lipase, bile, and calcium within a buffer solution. Unless otherwise stated, 0.5 g of each lipid source were incubated separately and in triplicate, with triplicate batch culture runs for each treatment in 40 mL of 0.5 M KH_2_PO_4_ (pH = 7.6) for 24 h with pancreatin (8 g/L), bovine bile (2.5 g/L), and CaCl_2_ (10 mM). Individually, concentrations of pancreatin, bile, and CaCl_2_, as well as incubation length were tested. To examine the use of this assay to estimate in vitro total tract digestion, a KH_2_PO_4_ solution with concentrated amounts to reach the same final concentrations of pancreatin, bile, and Ca were used as the third step in a three-step total tract digestibility procedure. Free glycerol and free fatty acid (FFA) concentrations were measured using colorimetric assays as indicators of digestion. Data wereanalyzed as a completely randomized block design (block = run), using the Glimmix procedure of SAS. For each lipid source, free glycerol increased with increasing pancreatin; however, FFA was lowest at 0 g/L pancreatin but was similar at 6, 8, and 10 g/L. Both glycerol and FFA were greater for 2.5 and 5 g/L of bile than for 0 g/L for each lipid source. Calcium concentration did not affect glycerol or FFA for either CO or CA; however, glycerol and FFA for BT were greater when calcium was included at 5 and 10 mM than at 0 mM. For all fat sources, free glycerol and FFA increased after 1 h until 12 h, but did not increase from 12 to 24 h. When a concentrated mixture was used following fermentation and acidification steps, digestibility using FFA concentration increased as compared to just adding buffer; however, free glycerol concentration was indeterminable. Thus, free glycerol and FFA can be used as indicators of lipid digestion when a lipid source is incubated for at least 12 h in a buffer solution containing 8 g/L pancreatin, 2.5 g/L bile, and 5 mM Ca when only estimating in vitro intestinal digestion; however, when utilizing this assay in a three-step in vitro total tract digestibility procedure, only FFA can be used.

## INTRODUCTION

For decades, rumen-protected feed additives have been available to cattle producers to supply their animals with an adequate source of amino acids, vitamins, or minerals without losing a significant portion to alteration by the microbial community of the rumen. Often times, these feed additives are coated with a lipid layer to prevent microbial degradation, which can lead to up to 95% rumen protection (Wu and Papas, 1997; Räisänen et al., 2020). However, once that feed additive passes out of the rumen, the protective coating must be digested in order for the nutrient inside to be available for absorption. While rumen degradability is easily estimated using in situ incubations, intestinal digestibility is much more difficult to evaluate. Digestibility trials, using either total collections or digesta markers to determine intestinal digestibility, are very invasive, costly and labor intensive and can take months to complete (Vanhatalo and Ketoja, 1994). Additionally, studying intestinal digestion can require the collection of intestinal digesta (Komarek, 1981) or employment of the mobile bag method (Voigt et al., 1985), both of which require surgery to cannulate the intestine. Furthermore, measuring individual lipid digestibility is difficult because animals consume complete diets rather than individual lipid sources. These trials also require animal approval protocols to perform invasive procedures such as ileum or duodenum cannulations that often affect animal welfare, reducing feed consumption and production, potentially making these animals unrepresentative of typical high-producing animals. Thus, an in vitro protocol to determine intestinal digestibility of lipids that is simple, practical, consistent, and repeatable would be a feasible solution.

Currently, in the ruminant nutrition industry, some work has been done to elucidate lipid digestion in vitro; however, there is no standardized in vitro protocol to determine lipid digestibility. The methodology described by Ross et al. (2013) includes lipase or pancreatin and could be used for lipid digestion, but was developed for the analysis of protein digestion, does not contain bile, and has not, to our knowledge, been validated for lipid digestion. This method has been further adapted by Zang et al. (2018) to include bile, but was only used to determine nitrogen digestion, thus still requires validation for its use in lipid digestion. There are however, several methods that have been used in human nutrition research (Garrett et al., 1999; Laurent et al., 2007; Hur et al., 2009, 2011). While humans have different digestive systems and diets compared to cattle, the basic components of lipid digestion in the small intestine are similar. Lipase, bile, and calcium concentrations have been investigated (Li et al., 2011), as have emulsifiers such as lecithin (Mun et al., 2007); so their interactions have been observed. However, the method of lipid digestion utilized in human nutrition laboratories is a regulated titration method that evaluates the rate of fatty acid release and requires specialized equipment (Li et al., 2011), making it difficult to adapt to a ruminant nutrition focus.

Calsamiglia and Stern (1995) developed the Three-Step method for determining protein digestibility in vitro. Their system uses a batch culture in vitro rumen fluid incubation (Goering and Van Soest, 1970) to determine ruminal degradation of protein sources, a mock abomasal acid-pepsin incubation, a simulated duodenal pancreatic enzyme digestion, and analysis of residual nitrogen to determine intestinal and total tract digestion of protein feeds. This method was modified by Ross et al. (2013) to eliminate the use of bags during digestion, which would be necessary to evaluate the digestion of liquid lipid sources. This assay provides the necessary steps to replicate digestion in vitro with the addition and manipulation of lipase, bile, calcium, and incubation length. We hypothesize that by manipulating the concentrations of lipase, bile, and calcium, as well as the incubation length in a three-step digestibility procedure, a simple, reliable assay for the determination of in vitro lipid digestion in cattle could be developed. Therefore, the objective of this study was to adapt the forementioned in vitro protocols to determine the extent of intestinal digestion of corn oil (CO), canola oil (CA), and beef tallow (BT) via manipulation of incubation length and concentrations of lipase, bile, and calcium within a buffer solution, as well as determining the efficacy of utilizing a three-step method to determine in vitro total tract digestion.

## MATERIALS AND METHODS

All animal care and handling procedures used in this experiment were approved by the University of Florida Institutional Animal Care and Use Committee.

### Materials

CO and CA (Publix brand; Publix Supermarket Inc., Lakeland, FL), and BT (EPIC Provisions, Austin, TX) were purchased from a local supermarket and were incubated without any further purification. These three lipid sources were chosen due to their use both currently and historically in the dairy industry, and the well-documented literature available regarding their digestibility (Jenkins, 2006) in order to compare the digestibility estimated in this experiment with those values reported from in vivo studies. The fatty acid composition (Table 1) of CO, CA, and BT were determined via AOAC Method 996.06 (AOAC, 2001) at the Chemical Laboratories at the Agricultural Experiment Station of the University of Missouri (Columbia, MO). Pancreatin (PP0010) was obtained from VWR (Radnor, PA) and is a combination pancreatic amylases, proteases, lipase, and colipase that is isolated only from porcine pancreas. Bile (bovine, B8381) was obtained from Sigma Aldrich Inc. (St. Louis, MO). Pancreatic lipase activity was 3 micro-equivalents of acid per minute and was determined using a commercial kit (kit number 700640; Cayman Chemical, Ann Arbor, MI). Calcium chloride (CaCl_2_ × 2H_2_O) was used to manipulate the concentration of calcium in solution (Li et al., 2011).

**Table 1.** The fatty acid composition (as % of DM) of corn oil (CO), canola oil (CA), and beef tallow (BT) used in the development of a small intestinal lipid digestion assay.

Fatty Acid	Treatment		
	CO	CA	BT
14:0	0.04	0.05	3.49
14:1 *c*-9	0.00	0.00	0.26
15:0	0.01	0.02	0.42
16:0	11.3	4.33	27.8
16:1 *c*-9	0.10	0.23	1.81
17:0	0.07	0.05	1.17
17:1 *c*-10	0.03	0.06	0.31
18:0	1.48	1.71	28.7
18:1 *t*-9	0.03	0.04	3.21
18:1 *c*-9	25.6	60.0	25.8
18:1 *c*-11	3.18	3.27	0.65
18:2 *t*-9, 12	0.05	0.00	0.14
18:2 *c*-9, 12	54.9	18.8	1.73
18:3 *c*-9, 12, 15	1.08	7.45	0.18
20:0	0.34	0.55	0.21
20:1 *c*-11	0.26	1.56	0.11
20:2	0.02	0.08	0.05
20:3 *c*-8, 11, 14	0.00	0.00	0.04
20:4 *c*-5, 8, 11, 14	0.00	0.00	0.02
21:0	0.01	0.06	0.04
22:0	0.06	0.27	0.04
22:1 *c*-13	0.00	0.02	0.00
22:2 *c*-13, 16	0.00	0.01	0.00
23:0	0.01	0.02	0.02
24:0	0.05	0.13	0.00
24:1 *c*-15	0.02	0.13	0.00

### Intestinal Digestion

Preparation of a 0.5 M potassium phosphate (KH_2_PO4) buffer solution was completed according to Calsamiglia and Stern (1995). Briefly, KH_2_PO_4_ was dissolved in deionized water and corrected to pH 7.6 using 5% NaOH. Concentrations of pancreatin, bile, and calcium as well as incubation length were all tested independently. However, unless otherwise stated, a standard solution of pancreatin (8 g/L), bile (2.5 g/L), and Ca (10 mM) was used, and samples were incubated at 39°C for 24 h (Calsamiglia and Stern, 1995) with constant agitation using a tilt table. Samples (0.5 g) of CO, CA, and BT were added to 150 mL Erlenmeyer flasks and incubated in 40 mL of buffer solution (Calsamiglia and Stern, 1995). The concentrations of this standard solution were determined during a pre-trial in which each variable was manipulated to find the range of each variable that needed to be tested. This standard solution and procedure were used to manipulate each variable individually without creating confounding effects. Dose–response analyses (Table 2) were conducted to determine the adequate concentrations of pancreatin (0, 6, 8, and 10 g/L), bile (0, 2.5, and 5 g/L), and calcium (0, 5, and 10 mM) and different incubation lengths (1, 3, 6, 9, 12, and 24 h) were investigated to determine the adequate length needed to achieve a level of digestion representative to that which is reported in vivo. Concentrations for pancreatin were selected based on concentrations used by Calsamiglia and Stern (1995). Concentrations of bile and calcium were selected based on the results of Li et al. (2011), in which they observed decreased digestion of CO beyond 5 g/L of bile and no change in digestion beyond 10 mM calcium. Final variable levels were selected based on a range of 70–80% digestibility of each lipid source to be comparable to the ranges of in vivo total tract digestibility for each source provided by Jenkins (2006) that were observed in lactating dairy cows.

**Table 2.** The variables and levels independently tested in the development of a small intestinal lipid digestion assay

Variable^1^	Range
Pancreatin^2^	0, 6, 8, and 10 g/L
Bile^3^	0, 2.5, and 5 g/L
Calcium^4^	0, 5, and 10 mM
Incubation length	1, 3, 6, 9, 12, and 24 h

^1^Variables were investigated independently in triplicate runs with three replicates per variable level per run.

^2^Pancreatin from porcine pancreas was obtained from VWR (PP0010; Radnor, PA).

^3^Bile was obtained from Sigma Aldrich Inc. (bovine, B8381; St. Louis, MO).

^4^Concentration of calcium was controlled using CaCl_2_.

Concentrations of pancreatin, bile, and calcium and incubation length were tested in separate sets of incubations arranged in a completely randomized block design. Each incubation was conducted using triplicate samples of each lipid source at each treatment level in three separate runs (block), for a total of *n* = 9 for each treatment level per lipid source. Different levels of treatment combination were not tested and each variable was investigated independently.

### Total Tract Digestion

After the final concentrations of pancreatin, bile, and Ca and the intestinal incubation length were determined, six replicated samples (0.5 g) of CO, CA, or BT were used in triplicated incubations for in vitro ruminal fermentation (Goering and Van Soest, 1970). Ruminal contents were collected from two mid-lactation, ruminally cannulated Holstein cows consuming a diet formulated to have a forage to concentrate ratio of 60:40. Contents were strained through four layers of cheesecloth into a pre-warmed thermos. After collection, contents were mixed with buffer and maintained at 39°C and bubbled with CO_2_. Serum bottles were flushed with CO_2_ and inoculated with 52 mL of buffered ruminal fluid and immediately closed and sealed and incubated at 39°C for 24 h.

Immediately following in vitro ruminal fermentation, serum bottles were opened and acidified with 2 mL of 3 M HCl (Ross et al., 2013) to simulate the action of the abomasum. Bottles were then capped and sealed and incubated at 39°C for 1 h. Immediately following incubation, serum bottles were opened and neutralized with 2 mL of 2 M NaOH (Ross et al., 2013).

Following neutralization, bottles were inoculated with 10 mL of a concentrated intestinal buffer solution (pH 7.6, 0.5 M KH_2_PO_4_, 16.5 g/L bile, and 9.64 g/L CaCl_2_) with or without pancreatin (52.8 g/L) in triplicate to determine the effects of ruminal fermentation on the extent of digestion. A smaller volume of concentrated buffer solution was used in this portion of the experiment in order to reach the same final concentrations of pancreatin, bile and CaCl_2_ as investigated in the previous portion of this experiment, as otherwise they would be diluted by the already present ruminal fluid and buffer solution. Bottles were mixed, re-sealed, and incubated at 39°C for 24 h. After 24 h, bottles were opened and 2 mL subsamples were collected and frozen at -20°C for later analysis of FFA concentrations.

### Estimation of Digestibility

The concentration of end products of triglyceride hydrolysis (free glycerol and fatty acids) was measured as estimates of digestibility. Free glycerol concentration was measured using a commercial kit (FG0100, Sigma Aldrich Inc., St. Louis, MO) and free fatty acid concentration was determined using the method described by Tinnikov and Boonstra (1999). Briefly, FFA were extracted using a chloroform-heptane solution and then mixed with a copper-triethanolamine reagent, which was then evaporated under nitrogen gas. An ethanol-diphenylcarbizide solution was then added and incubated for 15 min at ambient temperature prior to being read at 490 nm. Estimates of complete digestion (100% digestibility) were obtained by incubating triplicate samples of each lipid source in triplicated runs for 72 h with a pancreatin concentration of 16 g/L. Digestibilities of CO, CA, and BT were then calculated as a percentage of 100% digestibility of the respective lipid source.

### Statistical Analysis

Data for free glycerol and fatty acid estimates of digestibility were analyzed using the GLIMMIX procedure of SAS (SAS Institute, Cary, NC). Data from each lipid source were analyzed individually as a completely randomized block design, using run as a blocking factor with the following model:


$$\begin{array}{l} Y_{ij}=\mu +R_{i}+T_{j}+E_{ij}, \end{array}$$

In which *Y*_*ij*_ is the response variable, *µ* is the overall mean, *R* is the random effect of run (*j* = 1 to 3), *T* is the fixed effect of treatment (concentration of either pancreatin, bile, or calcium, the incubation length, or the presence or absence of pancreatin), and *E* is the residual error. Means separation was conducted using a Bonferroni test. Significance was declared at *P* ≤ 0.05 and tendencies were declared at 0.05 < *P* ≤ 0.10.

## RESULTS

### Pancreatin

**Figure 1.**
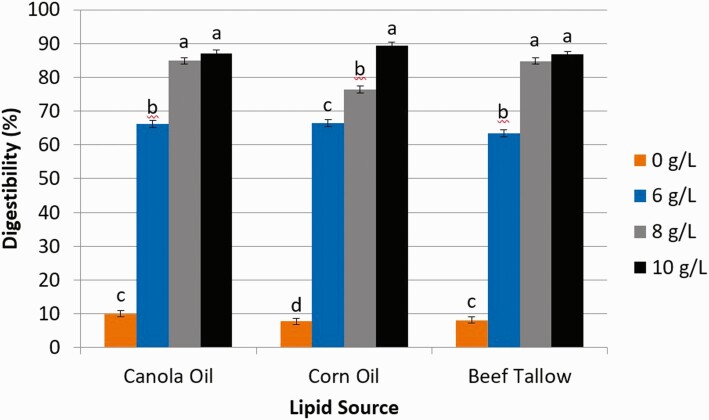
The effect of pancreatin concentration (0, 6, 8, and 10 g/L) on intestinal digestibility determined with free glycerol concentration released from either canola oil, corn oil, or beef tallow incubated for 24 h in a KH_2_PO_4_ buffer with 2.5 g/L of bile and 10 mM Ca. ^1^Significance declared at *P* ≤ 0.05. ^a-d^Different letters denote statistical difference.

**Figure 2.**
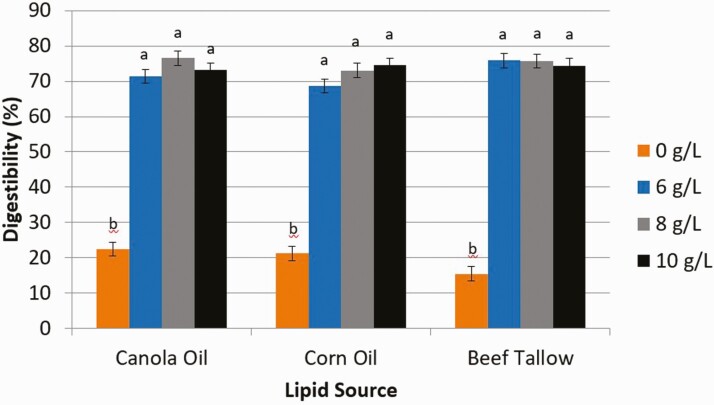
The effect of pancreatin concentration (0, 6, 8, and 10 g/L) on intestinal digestibility determined with free fatty acid concentration released from either canola oil, corn oil, or beef tallow incubated for 24 h in a KH_2_PO_4_ buffer with 2.5 g/L of bile and 10 mM Ca. ^1^Significance declared at *P* ≤ 0.05. ^a-b^Different letters denote statistical difference.

Digestibility estimated from the release of free glycerol (Figure 1) from CA and BT increased (*P* < 0.01) from 0 g/L (% digestibility for CA and BT, respectively; 10.0, 8.16) to 6 g/L (66.2, 63.5) and 8 g/L (84.9, 84.8), but were similar between 8 g/L and 10 g/L (87.0, 86.7) of pancreatin. However, digestibility (%) of CO increased (*P* < 0.01) as the concentration of pancreatin increased from 0 g/L (7.78), 6 g/L (66.42), 8 g/L (76.4), and 10 g/L (89.3). Digestibility estimated from the release of FFA (Figure 2) from CA, CO, and BT increased (*P* < 0.01) from 0 g/L of pancreatin (% digestibility for CA, CO, and BT, respectively; 22.5, 21.2, 15.5) to 6 g/L (71.3, 68.6, 75.8), but did not increase further with concentrations of 8 g/L (76.5, 73.1, 75.7) or 10 g/L (73.0, 74.6, 74.4).

### Bile

**Figure 3.**
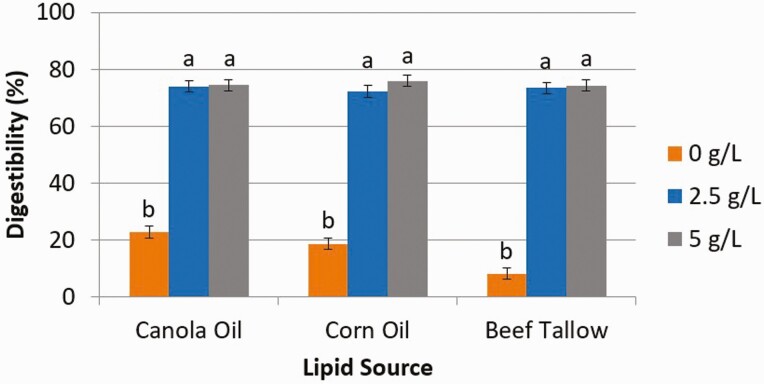
The effect of bile concentration (0, 2.5, or 5 g/L) on intestinal digestibility determined with free glycerol concentration released from either canola oil, corn oil, or beef tallow incubated for 24 h in a KH_2_PO_4_ buffer with 8 g/L of pancreatin and 10 mM Ca. ^1^Significance declared at *P* ≤ 0.05. ^a-b^Different letters denote statistical difference.

**Figure 4.**
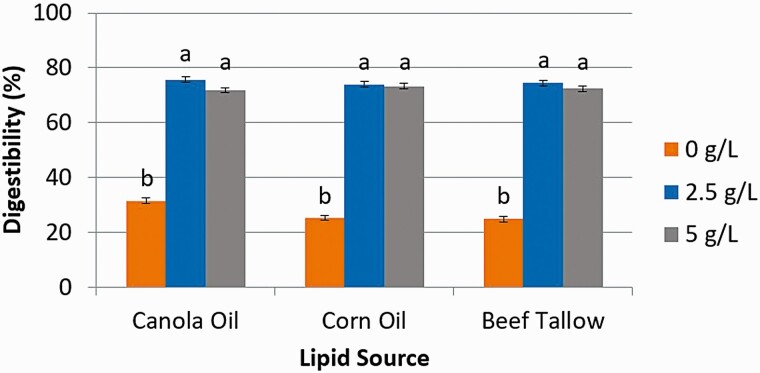
The effect of bile concentration (0, 2.5, or 5 g/L) on intestinal digestibility determined with free fatty acid concentration released from either canola oil, corn oil, or beef tallow incubated for 24 h in a KH_2_PO_4_ buffer with 8 g/L of pancreatin and 10 mM Ca. ^1^Significance declared at *P* ≤ 0.05. ^a-b^Different letters denote statistical difference.

The concentrations of bile used in this experiment were obtained from the results of Li et al., (2011) in which concentrations of bile greater than 5 g/L hindered in vitro digestion of CO. Digestibility estimated from the release of free glycerol (Figure 3) from CA, CO, and BT increased (*P* < 0.01) from 0 g/L (% digestibility for CA, CO, and BT, respectively; 22.7, 18.6, 8.08) of bile to 2.5 g/L (73.9, 72.2, 73.4) but was similar between 2.5 g/L and 5 g/L (74.4, 75.9, 74.2). Digestibility estimated from the release of FFA (Figure 4) from CA, CO, and BT increased (*P* < 0.01) from 0 g/L (% digestibility for CA, CO, and BT, respectively; 31.5, 25.2, 24.8) of bile to 2.5 g/L (75.6, 74.0, 74.4) but was similar between 2.5 g/L and 5 g/L (71.8, 73.3, 72.6).

### Calcium

**Figure 5.**
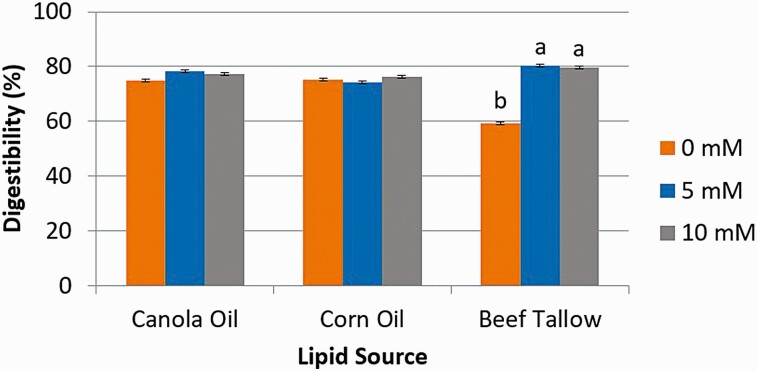
The effect of calcium concentration (0, 5, or 10 mM) on intestinal digestibility determined with free glycerol concentration released from either canola oil, corn oil, or beef tallow incubated for 24 h in a KH_2_PO_4_ buffer with 8 g/L of pancreatin and 2.5 g/L of bile. ^1^Significance declared at *P* ≤ 0.05. ^a-b^Different letters denote statistical difference.

**Figure 6.**
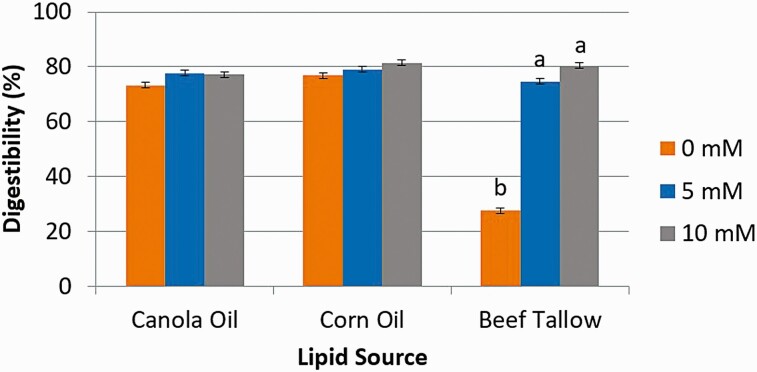
The effect of calcium concentration (0, 5, or 10 mM) on intestinal digestibility determined with free fatty acid concentration released from either canola oil, corn oil, or beef tallow incubated for 24 h in a KH_2_PO_4_ buffer with 8 g/L of pancreatin and 2.5 g/L of bile. ^1^Significance declared at *P* ≤ 0.05. ^a-b^Different letters denote statistical difference.

Digestibility estimated from the release of free glycerol (Figure 5) from CA and CO did not change with the addition of Ca (*P* > 0.05). However, the inclusion of Ca at either 5 mM (80.3%) or 10 mM (79.8%) increased (*P* < 0.01) the digestion of BT as compared to when Ca was absent (59.4%). Similar results were observed for digestibility estimated from the release of FFA (Figure 6) from CA and CO did not change with the addition of Ca (*P* > 0.05). However, the inclusion of Ca at either 5 mM (74.7%) or 10 mM (80.5%) increased (*P* < 0.01) the digestion of BT as compared to when Ca was absent (27.5%).

### Incubation Length

**Figure 7.**
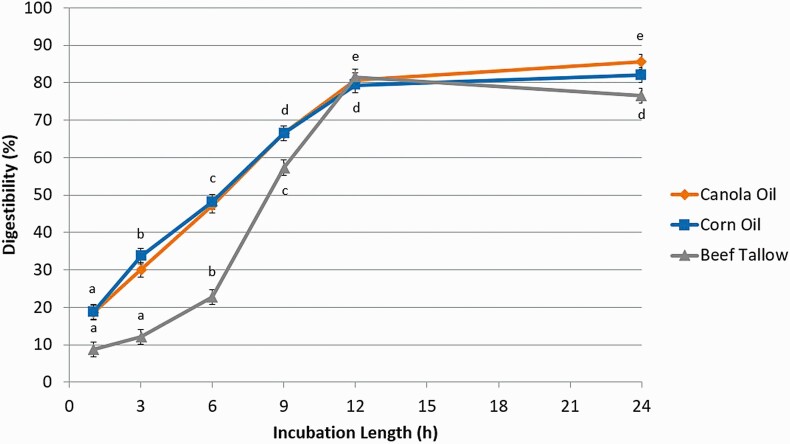
The effect of incubation length (1, 3, 6, 9, 12, or 24 h) on intestinal digestibility determined with free glycerol concentration released from either canola oil, corn oil, or beef tallow incubated in a KH_2_PO_4_ buffer with 8 g/L of pancreatin and 2.5 g/L of bile and 10 mM of Ca. Significance declared at *P* ≤ 0.05. ^a-e^Different letters denote statistical difference within each lipid source.

Digestibility of CA and CO determined with free glycerol release (Figure 7) increased (*P* < 0.01) between 1 h (% digestibility of CA and CO, respectively; 18.6, 18.9), 3 h (30.0, 33.7), 6 h (47.2, 48.1), 9 h (66.4, 66.5), and 12 h (80.7, 79.4), but was similar between 12 h and 24 h (85.6, 82.1). Digestibility of BT increased (*P* < 0.01) as well, but only between 3 h (12.1), 6 h (22.7), 9 h (57.3), and 12 h (81.5) and digestibility was similar between h 1 (8.72) and 3 and then h 12 and 24 (78.6).

**Figure 8.**
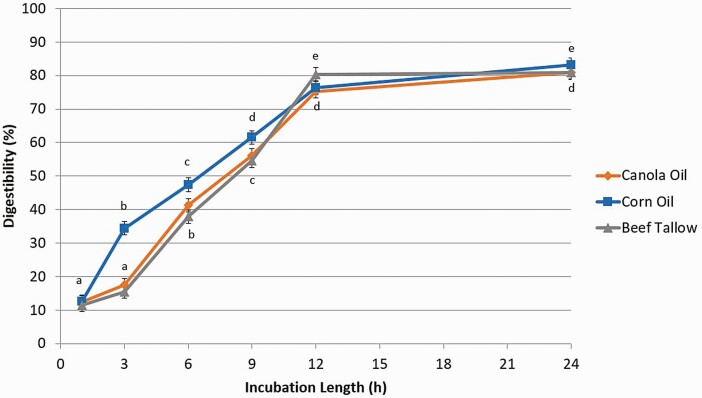
The effect of incubation length (1, 3, 6, 9, 12, or 24 h) on intestinal digestibility determined with free fatty acid concentration released from either canola oil, corn oil, or beef tallow incubated in a KH_2_PO_4_ buffer with 8 g/L of pancreatin and 2.5 g/L of bile and 10 mM of Ca. Significance declared at *P* ≤ 0.05. ^a-e^Different letters denote statistical difference within each lipid source.

Similar results were observed for the digestibility determined with FFA release (Figure 8) from CO. Digestibility increased (*P* < 0.01) between 1 h (% digestibility; 12.6), 3 h (34.4), 6 h (47.4), 9 h (61.5), and 12 h (76.3), but were similar between 12 h and 24 h (83.2). The pattern of digestibility of BT with FFA was similar to that which was observed with glycerol, and the pattern of FFA digestibility of CA was also similar to BT. Digestibility of CA and BT increased (*P* < 0.01) only between 3 h (% digestibility of CA and BT, respectively; 17.5, 15.5), 6 h (41.4, 37.9), 9 h (56.2, 54.5), and 12 h (75.3, 80.4) and digestibility was similar between h 1 (12.4, 11.5) and 3 and then h 12 and 24 (80.9, 81.0).

### Three-step Method

**Figure 9.**
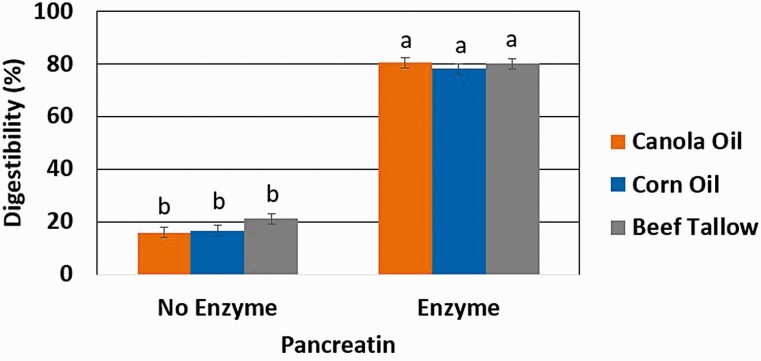
The effect of exclusion or inclusion of pancreatin (52.8 g/L) in a concentrated intestinal KH_2_PO_4_ buffer solution with 16.5 g/L of bile and 33 mM of Ca incubated for 24 h following a 24 h in vitro fermentation and 1 h mock abomasal digestion on the total tract digestibility as determined from free fatty acid release from either canola oil, corn oil, or beef tallow. ^1^Significance declared at *P* ≤ 0.05. ^a-b^Different letters denote statistical difference.

Digestibility of CA, CO, and BT as determined by the release of FFA (Figure 9) increased (*P* < 0.01) with the addition of a 24 h pancreatin incubation following both a 24 h in vitro ruminal fermentation and a 1 h acidic, mock abomasal digestion with 3 M HCl. Digestion of CA increased from 15.9% to 80.4%, CO from 16.6% to 78.1%, and BT from 21.1% to 80.1%. When determining digestibility of CA, CA, and BT using the full three-step in vitro digestibility method, only FFA was able to be used as an indicator of digestibility.

## Discussion

The objective of this study was to adapt existing in vitro digestibility methodologies to determine the necessary components for the determination of both total tract and intestinal lipid digestibility in vitro. Digestion of lipids in the small intestine is a multifaceted digestion that requires not only enzymatic action from lipase, but also requires bile as an emulsifier and calcium in order to remove freshly cleaved fatty acids from the surface of the lipid droplet (Holt, 1971; Brockman, 1984). In non-ruminant animals, the digestibility of nutrients is commonly determined by the differences in their content between feed and feces. However, in ruminants, microbial digestion in the rumen can alter the true intestinal digestibility of nutrients. While this can be remedied by the use of cannulated animals, digestibility trials can be expensive and labor intensive (Vanhatalo and Ketoja, 1994).

In the field of human nutrition, a common method to determine the in vitro digestibility of lipids is the pH-stat method (Ficara et al., 2003). This method is an automated titration that analyzes digestibility by measuring the change in pH in a buffer solution as a result of enzymatic release of negatively charged fatty acids as the lipid source is digested. While this method is both effective and efficient, it requires the use of expensive laboratory equipment; thus hindering the ability of this method to be widely used in ruminant nutrition laboratories. Therefore, we sought out to combine the assay developed by Calsamiglia and Stern (1995) and adapted by Ross et al. (2013) and the pH-stat method (Ficera et al., 2003). The adequate concentrations for pancreatin, bile, and Ca and the appropriate sample amount, buffer volume, and incubation length for both in vitro intestinal and total tract digestibilities are listed in Table 3.

**Table 3.** The proposed compositions of solutions used in in vitro intestinal digestions either as an individual procedure or as part of a three-step total tract in vitro digestibility procedure

Variable	Intestinal Digestibility	Total Tract Digestibility
Sample weight, g	0.5	0.5
Solution volume, mL	40	10
Pancreatin, g/L^1^	8	52.8
Bile, g/L^2^	2.5	16.5
Ca, mM^3^	5	33
Incubation length, h	≥ 12	≥ 12

^1^Pancreatin from porcine pancreas was obtained from VWR (PP0010; Radnor, PA).

^2^Bile was obtained from Sigma Aldrich Inc. (bovine, B8381; St. Louis, MO).

^3^Concentration of calcium was controlled using CaCl_2_.

### Pancreatin

The concentrations of pancreatin used in this experiment were obtained from Calsamiglia and Stern (1995), but the enzyme used in this experiment had a lower lipase activity than the one used in their experiment. Thus, based on pre-trial data, the concentrations of the pancreatin used in this experiment were doubled. While pancreatin contains enzymes unnecessary for the digestion of lipids (amylase and proteases), it also contains colipase. Therefore pancreatin was used in order to create a more replicable digestion of lipids as compared to an isolated lipase that does not include colipase.

Our results were similar to those obtained by Li et al. (2011), in which digestibility of CO increased with the increasing concentration of lipase. Additional lipase in solution allows for greater amounts of lipase molecules to be available for digestion of triglycerides at the oil–water interface of lipid droplets in solution. Lipase is a surface-active enzyme and is thus very dependent upon the surface area of the lipid droplet (Holt, 1971; Brockman, 1984). The lower digestibility values obtained using glycerol as an indicator of digestibility, as compared to the values of FFA digestibility, could be due to an incomplete digestion of some triglycerides in which FFA are released, but glycerol could still be bound to one or more fatty acids. Thus, based on our results, including pancreatin at 8 g/L of solution would provide a representative in vitro digestion of CA, CO, and BT; however, the use of FFA concentration as an indicator of digestibility may be more reliable than the concentration of free glycerol. This is because of instances of incomplete hydrolysis of triglycerides that leaves glycerol bound to one or more fatty acids and not detectable when determining free glycerol concentration.

### Bile

Bile is the main emulsifier of fat in the small intestine (Holt, 1971) and is responsible for the reduction of lipid droplet size within the intestinal lumen. Derived from cholesterol, bile is an amphiphilic compound that binds at the surface of the lipid droplet and can interact with the aqueous environment surrounding the lipid droplets (Holt, 1971; Brockman, 1984). This action will cause lipid droplets to decrease in volume and increase the surface area available for lipid hydrolysis by lipase (Drackley, 2000). Lipase is a surface-active enzyme and cannot penetrate the lipid droplet, thus it relies on access to fatty acids at the lipid–water interface of the droplet (Jones and Kubow, 2006). Therefore, the emulsification of lipids is imperative in their digestion.

Bile, in excess, has been reported to suppress lipase activity at the oil–water interface by blocking the hydrolysis of lipids by lipase (Brockman, 1984). Li et al. (2011) reported a decrease in both the rate and extent of digestion of CO when bile was included at 10 and 20 g/L of solution. They also reported no differences in rate nor extent of digestion when bile was included at either 2.5 or 5 g/L, similar to the results of this study. Thus, based on our results, in order to achieve an in vitro digestion representative of that observed in vivo, bile should be included at a concentration of 2.5 g/L of intestinal buffer solution.

### Calcium

Calcium concentrations were based on the results obtained by Hu et al. (2010) and Li et al. (2011). Both studies reported increased digestion of CO with the inclusion of calcium at 5 and 10 mM. Hu et al. (2010) reported an increase in digestion from 70% to 95% when calcium was increased from 10 to 20 mM; however, Li et al. (2011) reported no difference between 10 and 20 mM with a digestion of greater than 95%.

Both Hu et al. (2010) and Li et al. (2011) utilized the pH-stat method of in vitro digestibility and used an incubation length of 30 minutes. While both studies reported differences in CO digestibility with the addition of calcium; the present study utilizes a much longer incubation length, which could possibly explain the lack of an effect of calcium on CO digestibility. As mentioned previously, lipase is a surface-active enzyme (Jones and Kubow, 2006) and as lipids are digested, free fatty acids saturate the surface of the lipid droplet and are described as a shell around the lipid droplet by Li et al. (2011), preventing exposure to pancreatic lipase (Fave et al., 2004). Calcium acts to precipitate those cleaved FFA and remove them from the surface of the lipid droplet (Patton and Carey, 1979), thus acting as a cofactor for pancreatic lipase (Mukherjee, 2003) and allowing it to access the undigested portion of the lipid droplet. The differences in digestibility of BT with the addition of calcium could potentially be due to the greater concentrations of saturated FA in BT than in CO or CA. As CO and CA are both liquid at room temperature; their droplet size is visibly smaller in solution than BT, leading to a greater surface area on the CO and CA droplets for digestion than on the BT droplets (Brockman, 1984). Thus, the presence of calcium at a concentration of at least 5 mM is necessary in order to fully replicate the digestion of BT.

### Incubation Length

The delay in digestion between h 1 and h 3 for BT could be caused by the large droplet size of BT in solution preventing lipase from immediately accessing a large portion of the triglycerides (Brockman, 1984). However, there was an increase in the release of glycerol from CA between h 1 and h 3, but not FFA; thus it could have also been caused by fatty acids being bound and not immediately released from the droplet surface by lipase even though their glycerol had already been cleaved (Patton and Carey, 1979).

Given that for both free glycerol and FFA for all three lipid sources, there were no differences in digestibility between 12 h of incubation and 24 h; in order to achieve an in vitro digestion of lipids representative of what would be observed in vivo, a 12 h incubation is satisfactory. However, a 24 h incubation may be more convenient and can be used with no difference in digestibility.

### Three-step Method

While the focus of this manuscript was to design an assay to determine the in vitro intestinal digestibility of lipids, the potential for an assay to determine in vitro total tract digestibility had already been investigated by Calsamiglia and Stern (1995), Gargallo et al. (2006), and Ross et al. (2013). A potential use for this assay would be the estimation of digestibility of rumen-protected feed additives for both the level of protection as well as the intestinal availability of the additives. Rumen-protected feed additives that utilize lipids to prevent microbial fermentation in the rumen generally use a combination of triglycerides and saturated and unsaturated FA to either coat or embed the protected nutrient (Wu and Papas, 1997). Because of the use of FA in some rumen-protected feed additives, the use of free glycerol concentration as an indicator of digestibility is not feasible when evaluating these additives. While the determination of the level of rumen protection is relatively easy, the difficulty of determining intestinal availability could lead to the over or underestimation of the digestibility of rumen-protected feed additives using either total tract digestion via markers or total collection or using in situ methodology (Voigt et al., 1985). Overestimation could be detrimental to producers by either reducing the flow of available essential nutrients to the small intestine or increasing the amount of rumen-protected feed additives needed in order to reach the desired effects. On the other hand, underestimation could lead to the digestion of protected products in the rumen and reducing the true nutrient flow to the small intestine. Thus, the adaptation of the three-step procedures for in vitro digestion to determine lipid digestion was imperative to provide a less invasive method than in vivo cannulation methods and the mobile bag method (Voigt et al., 1985) and a less expensive method than large in vivo digestibility trials.

When glycerol was measured following incubation, it was not detected. This is possibly due to the fermentation of glycerol by ruminal bacteria present in solution (Garton et al., 1961). The fermentation of free glycerol by ruminal bacterial could lead to inaccurate estimation of digestibility. This could be further exacerbated by the use of FA without the presence of glycerol in some of the lipid encapsulated products and the partial hydrolysis of triglycerides. Thus, the concentration of FFA should be used as an indicator of digestibility instead of free glycerol.

## CONCLUSION

Representative digestion of CO, CA, and BT achieved in in vivo studies, can also be achieved utilizing an in vitro assay. In order to do so, we propose that a 0.5 g sample should be incubated in 40 mL of a 0.5 M KH_2_PO_4_ buffer solution with 8 g/L pancreatin, 2.5 g/L bile, and 5 mM Ca for at least 12 h. While glycerol and FFA are both end products of lipid digestion, FFA provide a more consistent result as an indicator of digestibility than free glycerol due to the potential for partially digested triglycerides. A similarly representative digestion can also be obtained by using a concentrated pancreatin mixture following a three-step in vitro total tract procedure; however, due to fermentation of glycerol, only FFA can be used as an indicator of digestibility. Further studies should consider evaluating other lipid sources, such as calcium soaps of fatty acids, as well as rumen-protecting products. Furthermore, in vivo validation would be needed to assess lipid utilization in the whole animal.

## References

[CIT0001] Brockman, H. L. 1984. General features of lipolysis: reaction scheme, interfacial structure and experimental approaches. In: B. Borgström and H. L. Brockman, editors, Lipases. Amsterdam: Elsevier Science Publishers B.V., pp. 3–46.

[CIT0002] Calsamiglia, S., and M. D. Stern. 1995. A three-step in vitro procedure for estimating intestinal digestion of protein in ruminants. J. Anim. Sci. 73:1459–1465. doi:10.2527/1995.7351459x. 7665377

[CIT0003] Drackley, J. D. 2000. Lipid metabolism. In: J. P. F. D’Mello, editor, Farm animal metabolism and nutrition. Wallingford, UK: CAB International Publishing. p. 97–119.

[CIT0004] Ficara, E., A. Rozzi, and P. Cortelezzi. 2003. Theory of pH-stat titration. Biotechnol. Bioeng. 82:28–37. doi:10.1002/bit.10541. 12569621

[CIT0005] Gargallo, S., S. Calsamiglia, and A. Ferret. 2006. Technical note: a modified three-step in vitro procedure to determine intestinal digestion of proteins. J. Anim. Sci. 84:2163–2167. doi:10.2527/jas.2004-704. 16864878

[CIT0006] Garrett, D. A., M. L. Failla, and R. J. Sarama. 1999. Development of an in vitro digestion method to assess carotenoid bioavailability from meals. J. Agric. Food Chem. 47:4301–4309. doi:10.1021/jf9903298. 10552806

[CIT0007] Garton, G. A., A. K. Lough, and E. Vioque. 1961. Glyceride hydrolysis and glycerol fermentation by sheep rumen contents. J. Gen. Microbiol. 25:215–225. doi:10.1099/00221287-25-2-215. 13703779

[CIT0008a] Goering, H. K., and P. J. Van Soest. 1970. Forage fiber analysis (Apparatus, Reagents, Procedures, and Some Applications). Agriculture Handbook No. 379. Washington (DC): ARS USDA.

[CIT0008] Holt, P. R. 1971. Fats and bile salts. J. Am. Diet Assoc. 60:491–498. 4554580

[CIT0009] Hu, M., Y. Li, E. A. Decker, and D. J. McClements. 2010. Role of calcium and calcium-binding agents on the lipase digestibility of emulsified lipids using an in vitro digestion model. Food Hydrocoll. 24:719–725.

[CIT0009a] Hur, S. J., B. O. Lim, G. B. Park, and S. T. Joo. 2009. Effects of various fiber additions on lipid digestion during in vitro digestion of beef patties. J. Food Sci. 74:C653–C657. doi:10.1111/j.1750-3841.2009.01344.x. 20492097

[CIT0010] Hur, S. J., B. O. Lim, E. A. Decker, and D. J. McClements. 2011. In vitro human digestion models for food applications. Food Chem. 125:1–12.

[CIT0011] Jenkins, T. C. 2006. Rendered products in ruminant nutrition. In: D. L. Meeker, editor, Essential rendering: all about the animal by-products industry. Alexandria (VA): National Renderers Association.

[CIT0012] Jones, P. J. H., and S. Kubow. 2006. Lipids, sterols, and their metabolites. In: M. E. Shils, M. Shike, A. C. Ross, B. Caballero, and R. Cousins, editors, Modern nutrition in health and disease. 10th ed. Baltimore (MD): Lippincott Williams and Wilkins. p. 92–122.

[CIT0013] Komarek, R. J. 1981. Intestinal cannulation of cattle and sheep with a T-shaped cannula designed for total digesta collection without externalizing digesta flow. J. Anim. Sci. 53:796–802. doi:10.2527/jas1981.533796x. 7319955

[CIT0014] Laurent, C., P. Besancon, and B. Caporiccio. 2007. Flavonoids from a grape seed extract interact with digestive secretions and intestinal cells as assessed in an in vitro digestion/Caco-2 cell culture model. Food Chem. 100:1704–1712.

[CIT0015] Li, Y., M. Hu, and D. J. McClements. 2011. Factors affecting lipase digestibility of emulsified lipids using an in vitro digestion model: proposal for a standardized pH-stat method. Food Chem. 126:498–505.

[CIT0016] Lim, J. B. O., E. A. Decker, and D. J. McClements. 2009. Effects of various fiber addition on lipid digestion during in vitro digestion of beef patties. J. Food Sci. 74:C653–C657. 20492097 10.1111/j.1750-3841.2009.01344.x

[CIT0017] Mukherjee, M. 2003. Human digestive and metabolic lipases a brief review. Journal Of Molecular Catalysis B: Enzymatic. 22:369–376.

[CIT0018] Mun, S., E. A. Decker, and D. J. McClements. 2007. Influence of emulsifier type on in vitro digestibility of lipid droplets by pancreatic lipase. Food Res. Inter. 40:770–781.

[CIT0019] Patton, J. S., and M. C. Carey. 1979. Watching fat digestion. Science 204:145–148. doi:10.1126/science.432636. 432636

[CIT0020] Räisänen, S. E., C. M. M. R. Martins, K. Nedelkov, J. Oh, M. T. Harper, A. Melgar, X. Chen, C. Parys, R. A. Patton, M. Miura, et al. 2020. Corrigendum to “Bioavailability of rumen-protected methionine, lysine and histidine assessed by fecal amino acid excretion”. Anim. Feed Sci. Technol. 268:114595.

[CIT0021] Ross, D. A., M. Gutierrez-Botero, and M. E. Van Amburgh. 2013. Development of an In-Vitro Intestinal Digestibility assay for Ruminant Feeds. Proceedings of the Cornell Nutrition Conference, 190–202.

[CIT0022] Tinnikov, A. A., and R. Boonstra. 1999. Colorimetric micro-determination of free fatty acids in plasma using microplate readers. Clin. Chim. Acta. 281:159–162. doi:10.1016/s0009-8981(98)00216-2. 10217636

[CIT0023] Vanhatalo, A., and E. Ketoja. 1994. The role of the large intestine in post-ruminal digestion of feeds as measured by the mobile-bag method in cattle. Br. J. Nutr. 73:491–505. doi:10.1079/bjn19950054. 7794867

[CIT0024] Voigt, J., B. Piatkowski, H. Engelmann, and E. Rudolph. 1985. Measurement of the postruminal digestibility of crude protein by the bag technique in cows. Arch. Tierernahr. 35:555–562. doi:10.1080/17450398509425219. 4074120

[CIT0025] Wu, Z., and A. Papas. 1997. Rumen-stable delivery systems. Adv. Drug Deliv. Rev. 28:323–334. 10837573 10.1016/s0169-409x(97)00087-2

[CIT0026] Zang, Y., S. S. Samii, W. A. Myers, H. R. Bailey, A. N. Davis, E. Grilli, and J. W. McFadden. 2018. Methyl donor supplementation suppresses the progression of liver lipid accumulation while modifying the plasma triacylglycerol lipidome in periparturient Holstein dairy cows. J. Dairy Sci. 102:1224–1236. doi:10.3168/jds.2018-14727. 30471914

